# Les accidents du travail dans le transport urbain en commun de la ville province de Kinshasa, République Démocratique du Congo: une étude transversale descriptive

**DOI:** 10.11604/pamj.2014.19.41.4020

**Published:** 2014-09-18

**Authors:** Jemima Wangata, Myriam Elenge, Christophe De Brouwer

**Affiliations:** 1 Centre de Recherche en Santé environnementale et Santé au travail, Ecole de Santé publique, Université libre de Bruxelles, Belgique

**Keywords:** Accident du travail, sécurité, secteur informel, transport urbain, Kinshasa, Occupational accident, security, informal sector, urban transit, Kinshasa

## Abstract

**Introduction:**

Le transport en commun urbain constitue un secteur où les travailleurs sont très exposés aux accidents du travail. Cette étude visait une description épidémiologique des accidents du travail dans le secteur informel du transport en commun à Kinshasa en vue d'apporter les pistes d'amélioration de la sécurité des travailleurs dans cette activité.

**Méthodes:**

Un questionnaire sur les accidents du travail, administré en Décembre 2012 a permis d'explorer les tendances significatives entre les accidents et leurs circonstances, leurs facteurs associés, leurs conséquences au sein d'une population des travailleurs (n = 472) du transport en commun à Kinshasa.

**Résultats:**

Durant les 12 derniers précédant l’étude 76.5% des travailleurs ont connu au moins un accident du travail, 54,8% ont connu un arrêt d'au moins 1jour. Les accidents liés à la circulation routière étaient plus important suivis des chutes. Les facteurs ayant montré des différences significatives étaient le travail sous l'influence de l'alcool et le port des équipements de protection individuelle. Les plaies (46,3%) et les contusions (39,4%) étaient les lésions les plus courantes. Les membres supérieurs (51,3%) et inférieurs (30,7%) étaient les plus atteints. 76,6% des travailleurs ont assumé seuls leur prise en charge médicale.

**Conclusion:**

L'incidence des accidents du travail dans ce secteur est très élevée. La mise en place d'une politique de prévention et gestion de différents facteurs associés ainsi qu'un système de déclaration d'accidents est nécessaire dans ce secteur. Les patrons ainsi que les politiques devraient veiller à une prise en charge médicale correcte pour des travailleurs accidentés.

## Introduction

Les accidents du travail figurent parmi les grands problèmes de santé publique dans le monde [1, 2]. Cependant, leurs données inégalement reparties dans différents secteurs d'activités, sont plus ou moins disponibles selon les pays [3]. Dans les pays industrialisés, les systèmes de déclaration obligatoire ainsi que les différents lois et règlements sur leur prévention ont permis de mieux suivre leurs données épidémiologiques [4, 5]. Dans les pays en voie développement par contre, selon les récentes recherches [6], les données officielles d'accidents de travail ne sont pas toujours basées sur des systèmes de notification appropriés [7]. De ce fait elles seraient inférieures à la réalité du problème parmi les populations des travailleurs. Cette étude s'est intéressée aux accidents du travail dans le secteur informel du transport en commun en République Démocratique du Congo plus précisément dans la ville province de Kinshasa.

Au cours de ces dernières années, dans ce pays, suite à la faillite de l’état dans le secteur transport en commun, ce service est dans une large mesure, assuré par les personnes privées, au sein d'un secteur informel. L'activité est pratiquée dans un contexte de survie et de précarité, sans le respect des normes de la circulation sur la voie publique et des mesures de sécurité pour les travailleurs. Cette situation est à l'origine de plusieurs accidents du travail. Compte tenue de l'importance du risque d′accidents du travail dans ce secteur, ainsi que du manque d′études épidémiologiques dans ce domaine, il a semblé indispensable d′améliorer nos connaissances sur ce problème de la sécurité des travailleurs.

Dans la suite d'une précédente recherche portant sur la santé des travailleurs au sein ce secteur [8], la présente étude descriptive s'est fixée comme objectif de produire des données épidémiologiques sur les accidents du travail au sein de cette activité, en termes d'incidence, facteurs associés et circonstances de survenue, de présenter leurs conséquences quant aux lésions physiques et arrêts de travail ainsi que les modalités de prise en charge médicale des travailleurs. Cette démarche avait comme but d'orienter les voies pour la mise en place des stratégies de prévention et de gestion de la sécurité des travailleurs dans ce secteur d'activité.

## Méthodes

### Population de l’étude et technique d’échantillonnage

Etude descriptive transversale à visée exploratoire sur un échantillon représentatif des travailleurs du secteur informel du transport en commun de la ville province de Kinshasa en République Démocratique du Congo. Les populations échantillonnées étaient constituées des chauffeurs et du personnel d'appoint membres de l'Association des Chauffeurs du Congo, ACCO (créée en 1979, elle constitue une des plus anciennes associations qui regroupe un grand nombre des travailleurs de ce secteur du transport). Les travailleurs étaient enregistrés au niveau des 24 parkings reconnus dans la ville de Kinshasa. Un échantillonnage aléatoire stratifié par le poste avec allocation proportionnelle a été pratiqué à partir d'un registre de près de 10.000 travailleurs. Les travailleurs étaient repartis en deux postes, notamment les chauffeurs dans une proportion de 40% ainsi que les membres du personnel d'appoint représentant la proportion de 60%. Les critères d'inclusion à l’étude étaient: avoir une ancienneté d'au moins une année au sein de l'activité et dans le même poste, avoir une carte de membre de l'ACCO, être volontaire pour répondre au questionnaire anonyme après un consentement éclairé. La taille d’échantillon a été calculée par le Logiciel Epi Info Version 7 (7.1.3). En acceptant une précision de 5% avec un niveau de confiance de 99%, il a été obtenu un d’échantillon de 528 individus. Sur cette taille obtenue, il a été alloué 40% aux chauffeurs soit 211 sujets. Cependant sur le terrain, seuls 184 chauffeurs ont effectivement répondu au questionnaire. Pour les membres du personnel, a été alloué les 60% sur la taille totale, soit 317 sujets. Seuls 288 ont pu répondre au questionnaire. Un total de 472 travailleurs dont 184 chauffeurs et 288 membres du personnel ont ainsi constitué l'effectif, soit un taux de réponse de 89,3%.

### Elaboration du questionnaire et collecte des données

L’élaboration du questionnaire s'est inspirée du modèle de la fondation européenne pour l'amélioration de la santé et conditions de travail [9]. Il a été soumis à la validation des pairs en santé et sécurité au travail de l'Ecole de Santé Publique de l'Université Libre de Bruxelles. Il comprenait trois catégories des questions notamment celles en relation avec les caractéristiques sociodémographiques, celles en rapport avec l'activité professionnelle, ainsi que les questions en rapport avec la survenue des accidents du travail et des problèmes de santé. Il a été traduit en lingala, la langue locale en vue d'une meilleure compréhension par les participants. Un pré-test a été réalisé en Novembre 2012. L'enquête proprement dite s'est déroulée en Décembre 2012. En rapport à l’éthique, le questionnaire était anonyme, l'administration auprès de chaque participant s'est effectuée après l'obtention d'un consentement éclairé.

### Méthodologie des analyses statistiques

Les données ont été encodées dans Excel 2007 et analysées dans le programme statistique SPSS, version 17.0. Initialement, une analyse descriptive a été réalisée pour décrire le profil de la population étudiée en fonction des caractéristiques sociodémographiques et des variables liées à l'activité. Cette description s'est effectuée par des analyses usuelles notamment les mesures de tendance centrale et de dispersion ainsi que la distribution des fréquences et des proportions. Le Chi carré de Pearson a été calculé. Afin de mieux interpréter les résultats de cette étude, certains variables ont été dichotomisées, créant deux groupes de comparaison au sein de l’échantillon. Il s'agissait du poste en distinguant le chauffeur et le personnel d'appoint. Pour l’âge, nous avons maintenue comme dans la précédente étude le seuil de 25 ans (qui constituait l’âge médian des travailleurs) en vue d'une meilleure comparabilité de certains résultats de deux études. Pour l'ancienneté le seuil fixé à 6 ans correspondait à la médiane pour cette variable dans l’échantillon. Pour ce qui est de l'alcool, ont été pris en compte deux types de variables à savoir, une simple consommation de l'alcool ainsi que le fait de travailler sous l'influence de l'alcool. La simple consommation d'alcool correspondait à une prise d'une à deux bouteille de bière de 72 Cl par jour (ce qui constitue la consommation moyenne dans la population générale à Kinshasa. Les bières locales sont dosées au taux de 6% d'alcool, nous avons utilisé ce taux pour catégoriser la simple consommation).

Pour le travail sous influence de l'alcool, le travailleur devait reconnaître une consommation d'alcool supérieure à deux bouteilles de bière par jour ou un abus d'alcool le conduisant dans un état d’ébriété partielle ou totale. En ce qui concerne les accidents du travail, il a été considéré comme accident, tout événement soudain, survenu du fait de l'activité, lors de l′exécution de la tâche, sur le lieu ou le chemin du travail. Pour la période de survenue, nous avons retenu les accidents ayant eu lieu durant les douze mois précédant l'enquête. Les accidents ont été distingués en deux groupes: premièrement les accidents de circulation ou du trafic routier survenant sur la voie publique impliquant au moins un véhicule et au moins une victime, secondairement les autres accidents non liés à la circulation routière. Parmi ces derniers, ont été distingués plusieurs circonstances d'accidents: les chutes en hauteur ou au même niveau, les glissades au niveau du sol, les accidents lors de la manutention des charges, les accidents par coincement des membres lors de l'ouverture et fermeture des portières du véhicule, les accidents par violences physiques par autrui, le corps étranger dans l’œil, et d'autres qui étaient non déterminés. L'incidence globale des accidents du travail au sein de la population a été calculée. Ont été déterminés les facteurs associés à la survenue des accidents. La proportion des travailleurs avec arrêt de travail d'au moins 1jour a été calculée, de même que les proportions pour chaque circonstance d'accidents.

## Résultats

### Description de l’échantillon

L'observation de la situation de travail a permis d'identifier dans le contexte de cette activité deux postes dont le chauffeur et le personnel d'appoint. Le chauffeur s'occupait de la conduite du véhicule, de l'organisation du matériel en relation avec le véhicule et parfois de la réparation du véhicule en cas de panne. Les membres du personnel d'appoint, souvent en dehors du véhicule pour l'exécution de leurs tâches respectives, s'occupaient de l'accueil du véhicule au niveau des parkings, du chargement des biens et des personnes, la vente des tickets ainsi que du contrôle des clients à la montée et descente du véhicule.

L'analyse sociodémographique de notre échantillon (Tableau 1) montre qu'il s'agit d'une activité exclusivement effectuée par les travailleurs du sexe masculin. L’âge médian était à 31 ans, la lecture des Percentile 5 et 95 a permis de noter que 90% de cette population étaient compris dans la tranche d’âge entre 22 et 42 ans.

**Tableau 1:** Description des variables socio-démographiques et variables d'activité

Variables	Répondants N= 472 (%)
Age (années)	
≤25	27,5
>25	72,5
**Alcool**	
Oui	81,8
Non	19,2
**Niveau d’étude**	
Aucune étude	12,5
Niveau bas	58,9
Niveau élevé	28,6
**Poste**	
Chauffeurs	39,9
Personnel d'appoint	60,1
**Formation**	
Oui	32,2
**Travail sous influence alcoolique**	
Oui	58,9
**Ancienneté**	
≤ 6	63,2
> 6	36,8
**Apprentissage**	
Oui	42,3
**Travail de nuit**	
Oui	62,5
**Travail de weekend**	
Oui	93,3

Il ressort du Tableau 1 que la consommation d'alcool est très élevée (81,8%) et plus de la moitié des travailleurs accomplissent leur tâche sous l'influence de l'alcool, dépassant parfois une consommation journalière de l’équivalent de 10 verres de bière. En outre, une large majorité (93,6%°) ont des horaires de nuit et plus de la moitié travaillent toute la semaine jusqu'au weekend end. En rapport à l'expérience, un peu plus de la moitié (62,5%) ont une ancienneté supérieure à 6 ans au sein du même poste. En termes de formation à la tâche, moins de la moitié (42,3%), notamment les chauffeurs ont reçu un apprentissage.

### Incidence des accidents

L'incidence globale était calculée à 76.5% pour N= 472. Soit 361 travailleurs ont connu au moins un accident du travail au cours des 12 derniers mois. Parmi ces 361 travailleurs, 71,7% ont déclaré avoir eu un arrêt de travail d'au moins 1 jour. Soit 54,9% d'arrêt de travail d'au moins 1jour sur tout l’échantillon.

### Analyse des variables associés aux accidents

**Figure 1:**
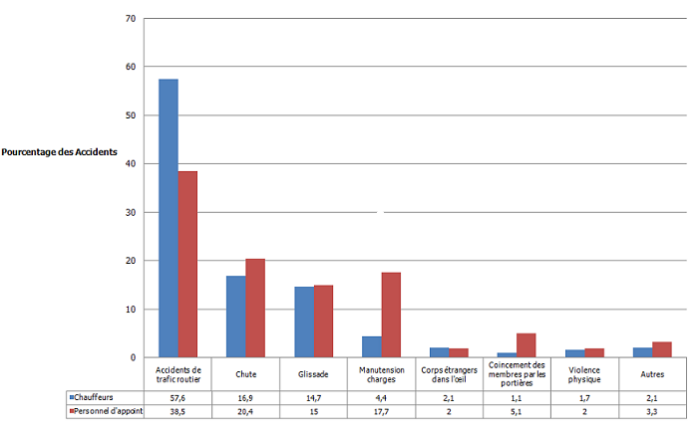
Circonstances de survenue des accidents du travail selon le poste

Parmi les circonstances de survenue présentées ci-dessous (Figure 1), les accidents de circulation routière avaient la plus grande proportion (57,6%) soit, plus de la moitié de l'ensemble des accidents notamment chez les chauffeurs. Ils étaient suivis de chutes et des glissades dont les proportions étaient beaucoup plus importantes parmi le personnel d'appoint. Les accidents survenant lors de la manutention des charges étaient dans la suite en importance soit 19,6% parmi les personnels d'appoint. Par contre les autres circonstances d'accidents restants, étaient de très faible proportion au sein de deux postes de travail.

En ce qui concerne les facteurs associés à la survenue des accidents indiqués ci- dessous (Tableau 2), les fréquences au sein des différents groupes de variables dichotomisés montrent que les facteurs tels que l’âge, l'ancienneté, l'apprentissage, le travail de nuit génèrent des différences pas assez significatives statistiquement en termes de fréquences d'accidents. Cependant, une différence nettement significative apparaît pour le port des équipements de protection individuels,EPI et l'influence alcoolique durant l'exercice de l'activité.

**Tableau 2:** Les déterminants des accidents parmi les sujets ayant connu les accidents

Variables	Répondants N= 361(%)	P
Age (années)		0,534
≤ 25	78,5	
>25	75,7	
**Ancienneté (années)**		0,274
≤ 6	74,8	
> 6	79,2	
**Apprentissage**		0,274
Oui	75,0	
Non	79,5	
**Travail sous influence d'alcool**		<0,001
Oui	27,5	
Non	2,5	
**Travail de nuit**		0,800
Oui	32,2	
Non	77,7	
**Travail de weekend**		0,008
Oui	77,8	
Non	56,7	
**Port d'EPI**		<0,001
Oui	24,9	
Non	75,1	

Sur les 361 travailleurs qui ont signalé avoir connu au moins un accident au cours de 12 derniers mois, plusieurs lésions ont été enregistrées, survenant les unes et les autres à la fois lors d'un événement accidentel. Parmi toutes ces lésions, un total de 1014 lésions reconnues chacune par le travailleur comme la lésion principale ou importante, ont été signalées. Ces lésions sont présentées dans cette suite (Tableau 3), groupées selon le type de dommage et la localisation. Il ressort de ce Tableau 3 que les plaies, suivies des contusions étaient les lésions les plus courantes et affectaient les membres supérieurs et inférieurs. Les fractures étaient de faible proportion. Nous précisons dorénavant à ce niveau que sur les 361 travailleurs accidentés, 349 ont reconnus avoir eu des lésions.

**Tableau 3:** Types et localisations des lésions considérées comme principales par les travailleurs lors la survenue des accidents

Localisation	Lésions (N= 1014) (%)				
	Plaies	Contusions	Entorses et Fractures	Brûlures	Total
Tête	5,2	4,2	-	0,8	10,2
Membres supérieurs	23,2	20,7	4,6	2,8	51,3
Membres inférieurs	15,1	10,9	3,2	1,5	30,7
Autres	2,8	3,6	0,8	0,6	7,8
Total	46,3	39,4	8,6	5,7	100

En fonction de la nature des soins, le Tableau 4 ci-dessous montre que 76,6% des travailleurs ayant déclaré une lésion ont bénéficié d'une prise en charge médicale dont les frais avaient été supportés par eux-mêmes ou leurs familles. Tenant compte de ces différents modes de prise en charge médicale, il apparaît également que 60,8% d'arrêts maladie de trois jours ou plus sont notés lorsque les soins sont pris en charge par le travailleur lui-même face à 3,8% d'arrêt maladie de même durée lorsque les soins sont pris en charge par le patron ou l'organisme d'assurance. Ces derniers soins étant supposés de qualité meilleure. Dans ce tableau le nombre des travailleurs est réduit à 349 du fait que parmi les 361 qui ont connu un accident, 12 n'ont pas déclaré de lésions.

**Tableau 4:** Importance de temps d'arrêt selon le type de prise en charge médicale

Type de Prise en charge des Médicale	Temps d'arrêt de travail (N= 349)%			Total
	Pas d'arrêt	Arrêt <3 jours	Arrêt ≥ 3 jours	
Prise en charge par le travailleur lui-même ou famille	12 ,1	3,7	60,8	76,6
Prise en charge par le patron ou la société d'assurance	16,2	3,4	3,8	23,4
Total	28,3	7,1	64 ,6	100

## Discussion

### Les biais de l’étude

Les biais éventuels de cette étude sont liés au « healthy work effect » [10]. L'absence des travailleurs malades lors de l'enquête constituerait un autre biais. Ce qui aurait réduit la prise en compte des cas d'accidents graves ayant entraîné le déclassement du travailleur concerné. Une réduction d'un éventuel biais de mémoire a été obtenue en limitant à une période de douze mois le temps de référence pour les questions en rapport avec les accidents du travail.

Quelques difficultés d'ordre organisationnel dans la mise en place de l'enquête nous ont conduits à considérer un seul mode de transport, qui reste le plus commun et accessible à toute la population dans cette ville. Il s'agit des taxis-bus utilisant les deux catégories de travailleurs, le chauffeur et son personnel d'appoint. Les résultats de cette étude ne peuvent s′appliquer immédiatement à tous les travailleurs dans les autres modalités de transport ce secteur notamment les motos taxis qui sont un nouveau mode de transport dans cette ville. D′autres études futures devraient s’étendre aux autres types de transporteurs.

### De l'importance des accidents du travail et circonstances de survenue

Cette étude révèle une incidence très élevée des accidents du travail (76,5%) au sein du secteur du transport en commun dans la ville de Kinshasa. Il est à noter que ces chiffres pourraient être sous estimés en raison de deux éléments majeurs que sont l'absence d'un système de déclaration d'accidents du travail officiellement établi ainsi qu'un certain défaut dans la perception de la notion d'accident du travail parmi ces travailleurs. En effet, à priori ceux-ci avaient tendance à retenir comme accidents du travail des événements qui auraient entraîné d'importantes lésions nécessitant une assistance médicale et ou conduisant à une incapacité de travail de longue durée. La notion d'accident du travail était souvent confondue avec celle du dommage. Les incidents ou presqu'accidents n'ayant entrainé que de légères lésions physiques avaient tendance à être méconnus. Lors du pré-test, la notion d'accident du travail a pu être clairement explicitée aux travailleurs. Au niveau national, en République Démocratique du Congo, nous n'avons pas trouvé d'autres études épidémiologiques sur les accidents du travail dans ce secteur informel du transport en commun. Nos résultats se rapprochent à ceux de Elenge [11] qui dans une étude sur les accidents de travail au sein d'une population des artisans miniers dans la province du Katanga a trouvé une incidence estimée à 72.2%. Ces artisans miniers exerçaient leurs activités dans un contexte de précarité des conditions de travail [12] plus ou moins comparable à celle de notre population d’étude, tel que cela a été présenté dans une autre étude [13] antérieurement réalisée au sein de cette activité, portant sur les indicateurs qualitatifs de la précarité.

Par contre dans une étude de Panda sur les accidents du travail dans le secteur formel notamment au sein d'une population des travailleurs d'une industrie textile [14] dans la province orientale de la République Démocratique du Congo, l'incidence des accidents de travail était de 60.2%. La précarité des conditions de travail au sein de ce secteur du transport en commun pourrait suffisamment expliquer ce taux d'incidence plus important dans la présente étude. En effet, dans la littérature certaines études [15, 16] ont lié la survenue des accidents aux mauvaises conditions de travail en tenant compte de certains facteurs comme de longs horaires de travail, une charge de travail trop élevée, le travail de nuit et de weekend end. Ces conditions sont décrites dans la population de la notre étude. Dans l’étude ci-haut citée, sur les indicateurs de la précarité au sein de ce secteur, cette activité professionnelle est décrite comme étant une option de survie importante en raison d'une certaine autonomie dans son exercice et de la possibilité raisonnable de la production des revenus.

L'analyse des circonstances de survenue de ces accidents permet de distinguer une première catégorie d'accidents, ceux liés à la circulation routière sur la voie publique ainsi qu'une seconde regroupant les autres circonstances d'accidents. Les accidents liés à la circulation routière présentent une tendance en proportion plus élevée que les autres circonstances d'accidents, et cela de manière plus prépondérante chez les chauffeurs. En effet, chez ceux-ci, dans notre échantillon, les accidents de circulation routière représentaient 56,7%, soit plus de la moitié de l'ensemble de tous les accidents. Des études réalisées dans d'autres pays notamment les pays développés démontrent que les efforts de prévention en matière de sécurité routière peuvent inverser cette tendance. Il s'agit notamment d'une étude réalisée au Danemark pour la période de 1993 à 2002 [17] dans le secteur du transport routier. Celle-ci a montré que les accidents liés à la circulation routière ne représentaient plus que moins d'un dixième de tous accidents de travail enregistrés chez les conducteurs de camion routier.

### De l'influence des facteurs sociodémographiques, personnels et professionnels

Dans notre étude, l'analyse des accidents en tenant compte des groupes d’âge n'a pas montré une différence statistiquement significative. Dans la littérature, la preuve de l′association entre l′âge et la fréquence des accidents du travail reste assez contradictoire. En effet, certaines études comme celle Hassen et al. [18] ont démontré que les travailleurs dans le secteur du transport qui ont subi un accident du travail étaient susceptibles d′être plus jeunes que les autres.

Par contre, l’étude de Dawson et al. [19] suggère que l’âge plus avancé serait un facteur prédictif de l'accident du travail. Ceci du fait que particulièrement, selon cette même étude, chez les travailleurs de transport, le vieillissement suggère des performances physiologiques dégradées résultant d'une altération de la vue et d'une modification de la physiologie de l′homéostasie du sommeil se traduisant souvent par la fatigue. Dans notre cas, l'absence de différence entre les travailleurs jeunes et les plus âgés pourrait s'expliquer par le fait que 90% de notre population d’étude était dans une même tranche d’âge, celle comprise entre 22 et 42 ans.

Quant à l'ancienneté, également dans notre échantillon, elle n'a pas montré une différence significative. Ces résultats ne confirment pas le rôle protecteur que pourrait procurer ce facteur dans la survenue des accidents, tel dans l’étude d'Elenge, ci haut citée selon [11]. Dans le cas de notre population des travailleurs, le manque d'effet protecteur chez les anciens travailleurs pourrait s'expliquer par une plus longue exposition aux conditions de travail précaires, reconnues par quelques études [20, 21] comme étant favorables à la survenue des accidents du travail. En ce qui concerne l'alcool, son usage n'a également pas montré une différence statistiquement significative dans la survenue des accidents au sein de notre échantillon. Par contre, ceux qui ont reconnu le fait de travailler sous l'influence de l'alcool avaient eu plus d'accidents que les autres, et la différence était significative. Plusieurs études réalisées dans le secteur du transport ont associé l'alcool à la survenue des accidents du travail, notamment ceux de la circulation routière [22–24]; dans le cas du travail sous l'influence de l'alcool, celui-ci selon la dernière étude, augmente le risque de survenue des accidents de par sa nature psycho active, pouvant modifier les perceptions et les comportements des travailleurs. Exum [25] a signalé une augmentation de l′agressivité et une diminution de la durée d′attention lors de la réalisation de la tâche.

Dans notre échantillon, parmi les autres circonstances, la violence physique était également décrite. Elle pourrait être attribuée à une plus grande agressivité des travailleurs en état d’ébriété. Parmi les autres facteurs, ceux qui poursuivaient l'activité jusqu'au weekend ont connu plus d'accidents en cette période que les autres, avec une différence statistiquement significative. En effet, une accumulation de la fatigue tout au long de la semaine pourrait expliquer cette situation, selon l’étude de Dawson et al., reprise plus haut [19]. L'apprentissage doit être également discuté, du fait qu'il n'a apporté aucune sécurité quant à la survenue des accidents du travail dans cet échantillon. En effet, le contenu de cet apprentissage est à remettre en question étant donné que cette activité s'exerce en dehors de la loi. Il faudrait également noter dans ce secteur, une certaine complaisance dans l'application stricte des règles professionnelles notamment dans la formation de ces travailleurs.

### De l'importance des lésions, la prise en charge et des temps d'arrêt de travail

La description des conséquences émanant des accidents dans ce secteur de transport permet de constater que les plaies et les contusions étaient les lésions plus importantes en termes de fréquence. Or en général par rapport aux brûlures et fractures qui étaient moins fréquents, elles sont de moindre gravité. Cette observation contraste avec la sévérité de ces accidents révélée par une proportion élevée des temps d'arrêt de travail de plus de trois jours, soit 64% des travailleurs. Ces faits peuvent s'expliquer par le « Healthy Worker Effect » [26], le fait que seuls les travailleurs sains étaient présents lors de l'enquête, ceux plus sévèrement atteints, en arrêt de travail, du fait des conséquences lésionnelles plus sévères auraient été absents lors de l'enquête. On pourrait par conséquent penser que les accidents invalidants auraient pu être nombreux, de même que les accidents mortels. D'autre part le fait qu'une grande majorité des accidents affectaient les membres supérieurs et inférieurs pourrait être la traduction d'une manipulation dangereuse des charges dans cette activité lors des efforts de manutention.

Cette activité se réalisant dans un contexte de précarité, il serait plus facile à comprendre qu'une proportion relativement importante des travailleurs soit 28,3% n'ait pas bénéficié d'un arrêt de travail et soit retourné à son poste après avoir connu un accident de travail. Ceci révèle suffisamment le besoin de survie qui régit cette activité. En effet les travailleurs victimes d'accidents devraient reprendre le travail le plus vite possible au dépens de leur santé, afin de subvenir à leurs besoins les plus élémentaires ou ceux de leurs familles. Le fait qu'une majorité de travailleurs ayant connu des lésions suites à leur accident, se prennent en charge eux-mêmes est un autre aspect traduisant cette précarité des conditions de travail au sein de cette population. Nous pensons que les cas pris en charge par le patron ou la société d'assurance étaient plus graves, nécessitant des soins plus spécialisés et donc exigeants nécessairement des frais plus importants. Ces dernières observations soulignent la nécessité d'une promotion de la santé et sécurité au travail au sein de ce secteur de transport en commun.

## Conclusion

Le travail dans le secteur du transport en commun des biens et des personnes a souvent été associé à un risque de survenue d'accidents du travail. Dans la présente étude l'incidence de ces accidents se relève cependant très élevée, ceci d'autant plus que ces travailleurs exercent leurs activités dans un contexte de précarité, le travail étant pour la plupart d'entre eux, un moyen de survie. Les accidents de circulation routière sur la voie publique avaient une fréquence plus élevée notamment chez les chauffeurs. Cela doit être pris en compte dans la mise en place d'une gestion des risques ainsi que de la prévention de ce problème de sécurité au travail dans cette population. De ce fait d'autres études plus ciblées sur ces accidents de circulation routière devraient en déterminer les facteurs associés de manière plus détaillée.

Les conditions de travail et d'emplois précaires dans un contexte informel demeurent un environnement qui alimente les problèmes de sécurité des travailleurs dans ce secteur et empêche l'application stricte de la règlementation en matière notamment d'alcool ou de la formation. Etant dans une activité de survie, cette application de la règlementation ne serait certes pas aisée, des formations ou vulgarisations aux changements d'habitudes et comportements des travailleurs pourraient être plus efficaces dans la gestion de ces accidents. Concernant leurs conséquences en termes de lésions et d'incapacité de travail, l'organisation d'une meilleure prise en charge médicale plus coordonnée semble être d'une grande nécessité. Une meilleure gestion des accidents du travail dans ce secteur dépendra en grande partie de l'amélioration dans l'organisation de l'emploi d'une part les associations des travailleurs eux-mêmes, de la responsabilisation des patrons ou propriétaires des véhicules par les politiques, quant à la prise en charge des problèmes de sécurité des travailleurs ainsi que leurs conséquences. Cette étude soulève par ailleurs la nécessité de la mise en place par les politiques d'un système de déclaration des accidents de travail dans ce secteur.
